# The glucose triad and its role in comprehensive glycaemic control: current status, future management

**DOI:** 10.1111/j.1742-1241.2010.02517.x

**Published:** 2010-11

**Authors:** A Ceriello

**Affiliations:** Insititut d'Investigacions Biomèdiques August Pi i Sunyer (IDIBAPS)Barcelona, Spain

## Abstract

The prevalence of type 2 diabetes across the world has been described as a global pandemic. Despite significant efforts to limit both the increase in the number of cases and the long-term impact on morbidity and mortality, the total number of people with diabetes is projected to continue to rise and most patients still fail to achieve adequate glycaemic control. Optimal management of type 2 diabetes requires an understanding of the relationships between glycosylated haemoglobin (HbA_1c_), fasting plasma glucose and postprandial glucose (the glucose triad), and how these change during development and progression of the disease. Early and sustained control of glycaemia remains important in the management of type 2 diabetes. The contribution of postprandial glucose levels to overall glycaemic control and the role of postprandial glucose targets in disease management are currently debated. However, many patients do not reach HbA_1C_ targets set according to published guidelines. As recent data suggest, if driving HbA_1C_ down to lower target levels is not the answer, what other factors involved in glucose homeostasis can or should be targeted? Has the time come to change the treatment paradigm to include awareness of the components of the glucose triad, the existence of glucose variability and their potential influence on the choice of pharmacological treatment? It is becomingly increasingly clear that physicians are likely to have to consider plasma glucose levels both after the overnight fast and after meals as well as the variability of glucose levels, in order to achieve optimal glycaemic control for each patient. When antidiabetic therapy is initiated, physicians may need to consider selection of agents that target both fasting and postprandial hyperglycaemia.

**Review Criteria**

A literature search was conducted based on expert direction and key words. A review of recent published data examining treatment targets and their impact on patients and the outcomes of large clinical trials conducted. Expert commentary on the key articles identified.

**Message for the Clinic**

As recent data suggest, if driving HbA_1c_ down to lower target levels is not the answer, what other factors involved in glucose homeostasis can or should be targeted? Has the time come to change the treatment paradigm to include awareness of the components of the glucose triad, the existence of glucose variability and their potential influence on the choice of pharmacological treatment? Understanding the underlying pathophysiological processes may help to improve overall management of patients with type 2 diabetes. When antidiabetic therapy is initiated, physicians may need to consider a selection of agents that target both fasting and postprandial hyperglycaemia.

## Introduction

Measurement of glycosylated haemoglobin (HbA_1c_) has been the focus of managing patients with type 2 diabetes for many years. Based on the outcomes of several landmark studies (1–4), guidelines for good glycaemic control have been agreed upon and a patient is generally considered to have achieved successful disease control when their HbA_1C_ is < 7% (5–7). Management of the patient with type 2 diabetes requires continuous monitoring and currently this may involve occasional measurement of fasting plasma glucose as an indicator of the efficiency of the body in regulating glucose levels in the absence of dietary glucose. However, better understanding of the pathophysiology underlying type 2 diabetes has indicated that control of fasting plasma glucose levels is not critical in early stage disease (8). In addition, fasting plasma glucose does not correlate well with HbA_1C_ (9,10), suggesting that there may be other factors that make a significant contribution to overall glycaemic control. Recent evidence (11) has highlighted the role of postprandial glucose levels and associated glycaemic variability in achieving and maintaining comprehensive glycaemic control in patients with type 2 diabetes.

## Changing the paradigm

Over the years, target HbA_1C_ levels have been the subject of much debate, but until recently, it has been accepted that HbA_1C_ should be as low as is realistically achievable. The strategy of ‘the lower, the better’ was reinforced by data from the UK Prospective Diabetes Study (UKPDS) that showed that any reduction in HbA_1C_ in patients with type 2 diabetes is likely to reduce the risk of complications, with the lowest risk being in those with HbA_1C_ values < 6% (1).

However, more recent studies have raised concerns that intensive treatment and stringent HbA_1C_ targets may be detrimental in some patients. The Action to Control Cardiovascular Risk in Diabetes (ACCORD) trial was stopped early when it was found that there was an increased risk of death in patients who received intensive blood glucose-lowering therapy with an HbA_1C_ target of < 6% (12). Patients who experienced severe hypoglycaemia were at increased risk of death regardless of whether they were receiving intensive or standard treatment (13). Moreover, both ACCORD (12) (target HbA_1C_ < 6%) and the Action in Diabetes and Vascular Disease: Preterax and Diamicron Modified Release Controlled Evaluation (ADVANCE) (14) (target HbA_1C_ < 6.5%) trials failed to show that achievement of good glycaemic control was associated with reduction of cardiovascular risk. These findings appear to be supported by results from a new retrospective cohort study that was conducted in the UK (15). Patients with type 2 diabetes whose glucose-lowering treatment had been intensified were identified from general practitioner records. Low and high HbA_1C_ levels were associated with increased mortality and cardiac events, with the lowest risk seen at an intermediate HbA_1C_ of 7.5%. This study did have several limitations, including failure to take into account concomitant therapy for cardiovascular disease, non-standardised measurement of HbA_1C_ and missing data. In addition, the study was conducted in the UK where general practitioners are encouraged to pay more attention to patients with HbA_1C_ > 7% than to those who are better controlled. Nevertheless, the study has contributed further to the current debate and discussion.

**Figure 1:**
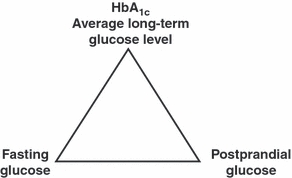
HbA_1C_, postprandial glucose and fasting plasma glucose interrelate and are essential targets for intervention in attempts to optimise overall glycaemic control. This figure was published in Diabetes and Metabolism; 32: Special Issue no 2. Monnier L, Colette C, Boniface H, Contribution of postprandial glucose to chronic hyperglycaemia: from the “glucose triad” to the trilogy of “sevens”. 2S11–2S16, Copyright Elsevier 2006

Most patients with type 2 diabetes are still failing to achieve adequate glycaemic control and the disease remains a major cause of morbidity and mortality (16–19). But the conundrum remains: if driving HbA_1C_ down to lower target levels is not the answer, what other factors involved in glucose homeostasis can or should be targeted? For several years, the related phenomena of daily plasma glucose variability and postprandial glucose levels have been under scrutiny, particularly in relation to HbA_1C_ and fasting plasma glucose. Although their position in the so-called glucose triad is gaining acceptance (Figure 1) (20–23), there is ongoing debate regarding the contribution of postprandial glucose levels to overall glycaemic control and the role of postprandial glucose targets in the management of a patient with type 2 diabetes.

## Comprehensive glycaemic control – the role of postprandial glucose and glucose variability

**Figure 2:**
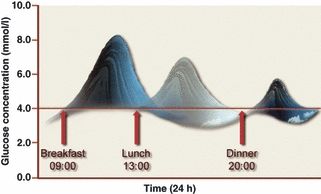
Blood glucose profile over 24 h in an individual with type 2 diabetes

In individuals with normal glucose tolerance, the plasma glucose concentrations generally rise no higher than 7.8 mmol/l after a meal and return to normal levels within 2–3 h. In contrast, in individuals with type 2 diabetes, postprandial plasma glucose levels > 7.8 mmol/l are common, even in those who are considered to have good overall glycaemic control according to measurement of HbA_1C_ (Figure 2). In fact, achievement of target HbA_1C_ and fasting plasma glucose levels does not necessarily indicate that good glycaemic control is continuous throughout the day.

**Figure 3:**
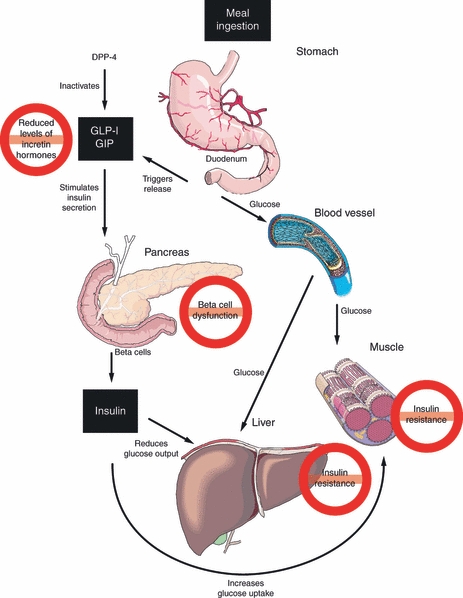
The role of GLP-1 and GIP in glucose homeostasis. Key defects in individuals with type 2 diabetes are shown in red circles

Type 2 diabetes is a progressive disease. The typical course is initially a gradual loss of glycaemic control after meals, followed by the development of fasting hyperglycaemia in the morning and finally sustained hyperglycaemia during the night. Patients who have impaired glucose tolerance, but have not yet developed type 2 diabetes tend to have near normal fasting plasma glucose, but show variable glucose excursions after the three meals of the day (24). The key pathological effect at this prediabetes stage is loss of first phase insulin secretion. This is the early surge of insulin that occurs within 5 min of eating and is critical for suppression of hepatic glucose production and priming the liver and peripheral tissues, particularly muscle and fat, for glucose uptake (Figure 3).

The onset of frank type 2 diabetes is characterised by a progressive decline in insulin sensitivity together with progressive deterioration in beta-cell function leading to reduced insulin secretion. Increased fasting plasma glucose levels in patients with type 2 diabetes are largely attributable to reduced hepatic sensitivity to insulin leading to overproduction of glucose by the liver during the overnight fast (25). As diabetes progresses, these effects persist into the morning and result in particularly marked hyperglycaemia following breakfast (8).

In contrast to fasting hyperglycaemia, the causes of postprandial hyperglycaemia are much more complex. Postprandial glucose levels are influenced by the blood glucose level before the meal and the glucose load from the meal, as well as physiological factors such as insulin secretion and insulin sensitivity in the peripheral tissues. The incretin hormones, glucagon-like peptide (GLP)-1 (Table 1) (26) and gastric inhibitory polypeptide (GIP) are released by the intestine in response to ingestion of carbohydrate. These hormones enhance insulin secretion, suppress hepatic glucose production and decrease gastric emptying and have a greater effect on postprandial glucose levels than fasting glucose levels. Patients with type 2 diabetes have reduced levels of the incretin hormones.

**Table 1:** Actions of glucagon-like peptide 1 (26)

Brain	Induces feeling of satiety
	Reduces food intake
Gastro intestinal tract	Delays gastric emptying
	Delays food absorption
Pancreas	Stimulates glucose-dependent insulin secretion
	Suppresses glucagon secretion
	Increases beta-cell sensitivity
	Increases beta-cell mass (animal studies only)
Liver	Decreases hepatic glucose output because of reduced glucagon secretion
Fat/muscle	Stimulates glucose uptake
Heart	Increases myocardial protection
	Improves endothelial function
	Decreases blood pressure
	Improves left ventricular function

**Figure 4:**
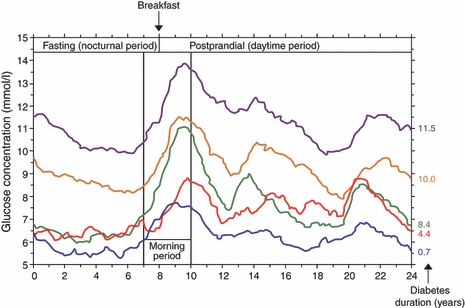
The 24-h recordings from a continuous glucose monitoring system in five groups of patients with type 2 diabetes. Blue: HbA_1C_ < 6.5%; red: ≥ 6.5% to < 7%; green: ≥ 7% to < 8%; orange: ≥ 8% to < 9%; purple: ≥ 9%. Reproduced with permission from Monnier L et al. Diabetes Care 2007;30:263–9

It is important to understand the relationships between HbA_1C_, and fasting and postprandial blood glucose, and how these change during progression of the disease, if type 2 diabetes is to be managed optimally. Fasting and postprandial plasma glucose both contribute to HbA_1C_. However, the relative contribution of these two factors depends on the HbA_1C_ level, with postprandial glucose contributing relatively more at lower HbA_1C_ levels (27,28). Early in the course of the disease, when fasting plasma glucose levels are near normal, postprandial glucose is more important in determining HbA_1C_. Measurement of 24-h plasma glucose profiles in patients with HbA_1C_ of < 6.5%, ≥ 6.5% to < 7% and ≥ 7% to < 8% showed that fasting plasma glucose levels were very similar in these three groups, with the principal difference being in postprandial glucose (Figure 4) (8). These data suggest that reduction of HbA_1C_ in patients who are close to target (< 8%) is best achieved by specifically targeting postprandial glucose levels. As glucose control deteriorates and HbA_1C_ rises, the contribution of fasting plasma glucose becomes more significant. In groups of patients with HbA_1C_ of ≥ 8% to < 9% and ≥ 9%, fasting plasma glucose progressively increased, indicating that control of *both* fasting and postprandial glucose is important at these higher HbA_1C_ levels (Figure 4) (8).

**Figure 5:**
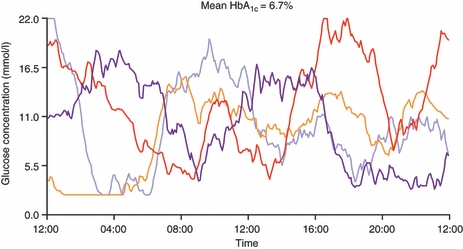
Individual 24-h recordings from a continuous glucose monitoring system in four patients with type 2 diabetes on insulin therapy and a mean HbA_1C_ of 6.7%

Short-term glucose fluctuations or spikes may also have important clinical implications. Plasma glucose excursions following a meal are generally greater, last longer and are more variable in patients with type 2 diabetes compared with the normal population (Figure 5). A recent review of all available evidence suggested that variability in plasma glucose levels may be an independent risk factor for the development of microvascular and macrovascular complications and mortality (29). Smoothing the daily glucose profile by reducing the amplitude of glucose spikes may result in improved overall glycaemic control and thus a theoretical reduction in the associated complications.

## Potential adverse effects of elevated postprandial glucose

The specific relationship between postprandial hyperglycaemia and the development of diabetic complications is unclear. Postprandial hyperglycaemia does appear to be correlated with the risk of microvascular complications (11). There is also some evidence suggesting that raised postprandial glucose may be an independent risk factor for macrovascular complications, particularly for cardiovascular disease, but as this is still the subject of intensive research, no definitive conclusions can be drawn (30–35).

Epidemiological data suggest that postprandial hyperglycaemia is a risk factor for the development of cardiovascular diseases, but there remains a need for evidence that lowering postprandial hyperglycaemia will help prevent cardiovascular disease. Results from the Study to Prevent Non-Insulin-Dependent Diabetes Mellitus (STOP-NIDDM) trial, in which the impact of postprandial hyperglycaemia was evaluated as a predefined secondary end point, suggest that treating postprandial hyperglycaemia may reduce the incidence of new cardiovascular events in people with impaired glucose tolerance (36). This finding was supported by a meta-analysis on the use of acarbose in patients with type 2 diabetes (37). However, the Hyperglycaemia and its Effect after Acute Myocardial Infarction on Cardiovascular Outcomes in Patients With Type 2 Diabetes (HEART2D) study (35) and the Nateglinide and Valsartan in Impaired Glucose Tolerance Outcomes Research (NAVIGATOR) study in those with impaired glucose tolerance (38) both failed to confirm this finding. The HEART2D study did not reach the predetermined difference in postprandial blood glucose of 2.5 mmol/l between patients randomly assigned to prandial or basal strategies; the mean difference between the two groups at the end of the study was 0.8 mmol/l, less than one-third of the goal, even though the difference was significant (35). In the NAVIGATOR trial, not only did nateglinide not improve postprandial hyperglycaemia, but glucose levels 2 h after an oral glucose challenge were higher in the nateglinide group than in the placebo group (38). Furthermore, the incidence of new diabetes was slightly higher in the nateglinide-treated group than in the placebo group (36.0% vs. 33.9%) – although this was not statistically significant – and nateglinide also increased the risk of hypoglycaemia.

There were, however, a number of potentially confounding factors in the NAVIGATOR study, which mean that these results have only added to the ongoing debate. It is important to be aware of the level of cardiovascular risk of patients included in clinical studies (33). Although data suggest that the control of hyperglycaemia may have a different impact on primary and secondary prevention of cardiovascular disease in patients with type 2 diabetes, in the NAVIGATOR study, patients in these two groups were pooled and evaluated together (38). A further concern is the very high dropout rate.

What does seem to be more certain, however, as recent lessons from ACCORD (39,40), ADVANCE (41), Veteran’s Affairs Diabetes Trial (VADT) (42) and from the long-term follow-up of the UKPDS (43) suggest, is that if the control of hyperglycaemia, whether fasting (ACCORD, ADVANCE, VADT) or postprandial (35), is started too late, the possible beneficial effect of treatment that is initiated in a very early stage of the disease is lost (2,36).

## Impact of HbA_1c_, fasting plasma glucose and postprandial glucose on management approaches and treatment choice

Early and sustained control of glycaemia is important in the management of type 2 diabetes. Many patients do not reach HbA_1C_ targets set according to published guidelines (16–19). Following publication of ACCORD, ADVANCE and other studies (12,15,44), management guidelines are moving towards a recommendation that it is more appropriate to agree upon individual goals with each patient taking into account age, comorbidity, personal circumstances and attitudes, etc. (45). Regardless of the HbA_1C_ goal that is agreed upon, it is unlikely to be reached unless both fasting and postprandial glucose levels are adequately controlled, ideally through a combination of lifestyle modification and appropriate drug therapy.

All people who are living with diabetes should be given information and education that are tailored to their individual needs. Lifestyle modifications are an important part of the treatment plan and can also help to reduce postprandial hyperglycaemia. In particular, altering the quantity and composition of the meal and taking regular exercise can be beneficial (46). Foods with a lower glycaemic index contain carbohydrates that are more slowly digested and absorbed. There is some evidence that diets with a low glycaemic load are beneficial in reducing postprandial glucose excursions (47).

Routine measurement of postprandial glucose levels is not currently recommended or even practical for all patients with type 2 diabetes. However, improved understanding of the relative influence of fasting and postprandial glucose levels throughout the course of the disease might influence the class of drug that is prescribed. Recent research has suggested that intensification of glucose control with insulin therapy may not be advisable for all patients with type 2 diabetes and oral antidiabetic drugs should be used for as long as possible (15). International Diabetes Federation (IDF) guidelines for the management of postmeal (postprandial) glucose state that the goal of diabetes therapy should be to achieve glycaemic status as near to normal as safely possible in all three measures of glycaemic control, namely HbA_1C_, fasting premeal glucose and postmeal glucose (47). Treatment of both fasting and postprandial hyperglycaemia should be initiated simultaneously at all levels of HbA_1C_ above agreed levels. Traditional treatments such as metformin and thiazolidinediones primarily lower fasting plasma glucose. As sulphonylureas are generally taken in the morning, they do lower postprandial glucose levels during the day and subsequently have an effect on overnight fasting levels. Therapeutic agents are available that preferentially lower postprandial glucose, including alpha-glucosidase inhibitors, glinides, incretin mimetics, dipeptidyl peptidase (DPP)-4 inhibitors and rapid-acting insulins. An ideal approach to the treatment of a patient with newly diagnosed type 2 diabetes might be to start with the combination of metformin and a DPP-4 inhibitor. This combination effectively targets the two key pathophysiological features of type 2 diabetes: loss of first phase insulin secretion and insulin resistance. Combination of a DPP-4 inhibitor with metformin is likely to be better tolerated than combination with a sulphonylurea, with a lower incidence of weight gain and a very low risk of hypoglycaemia (48).

## Conclusions

It is becomingly increasingly clear that physicians are likely to have to consider plasma glucose levels both after the overnight fast and after meals in order to achieve optimal glycaemic control for each patient. The optimal glycaemic control equation equates to HbA_1C_ (at target) + fasting plasma glucose (to target) + postprandial glucose (to target) without hypoglycaemia and weight gain. Although target HbA_1C_ levels can be reached by lifestyle modification together with combination drug therapy, optimal glycaemic control may be best achieved by selection of agents that target both fasting and postprandial hyperglycaemia.
